# A Cross-Reactive Monoclonal Antibody to Nematode Haemoglobin Enhances Protective Immune Responses to *Nippostrongylus brasiliensis*

**DOI:** 10.1371/journal.pntd.0002395

**Published:** 2013-08-29

**Authors:** Natalie E. Nieuwenhuizen, Jeanne M. Meter, William G. Horsnell, J. Claire Hoving, Lizette Fick, Michael F. Sharp, Matthew G. Darby, Suraj P. Parihar, Frank Brombacher, Andreas L. Lopata

**Affiliations:** 1 International Center for Genetic Engineering and Biotechnology (ICGEB), Cape Town Component, and Institute of Infectious Diseases and Molecular Medicine (IIDMM) and Medical Research Council, Division of Immunology, Faculty of Health Science, University of Cape Town, Cape Town, South Africa; 2 School of Pharmacy and Molecular Science, Centre for Biodiscovery and Molecular Development of Therapeutics, James Cook University, Townsville, Queensland, Australia; McGill University, Canada

## Abstract

**Background:**

Nematode secreted haemoglobins have unusually high affinity for oxygen and possess nitric oxide deoxygenase, and catalase activity thought to be important in protection against host immune responses to infection. In this study, we generated a monoclonal antibody (48Eg) against haemoglobin of the nematode *Anisakis pegreffii*, and aimed to characterize cross-reactivity of 4E8g against haemoglobins of different nematodes and its potential to mediate protective immunity against a murine hookworm infection.

**Methodology/Principal Findings:**

Immunoprecipitation was used to isolate the 4E8g-binding antigen in *Anisakis* and *Ascaris* extracts, which were identified as haemoglobins by peptide mass fingerprinting and MS/MS. Immunological cross-reactivity was also demonstrated with haemoglobin of the rodent hookworm *N. brasiliensis*. Immunogenicity of nematode haemoglobin in mice and humans was tested by immunoblotting. *Anisakis* haemoglobin was recognized by IgG and IgE antibodies of *Anisakis*-infected mice, while *Ascaris* haemoglobin was recognized by IgG but not IgE antibodies in mouse and human sera. Sequencing of *Anisakis* haemoglobin revealed high similarity to haemoglobin of a related marine nematode, *Psuedoterranova decipiens*, which lacks the four –HKEE repeats of *Ascaris* haemoglobin important in octamer assembly. The localization of haemoglobin in the different parasites was examined by immunohistochemistry and associated with the excretory-secretary ducts in *Anisakis*, *Ascaris* and *N. brasiliensis*. *Anisakis* haemoglobin was strongly expressed in the L3 stage, unlike *Ascaris* haemoglobin, which is reportedly mainly expressed in adult worms. Passive immunization of mice with 4E8g prior to infection with *N. brasiliensis* enhanced protective Th2 immunity and led to a significant decrease in worm burdens.

**Conclusion:**

The monoclonal antibody 4E8g targets haemoglobin in broadly equivalent anatomical locations in parasitic nematodes and enhances host immunity to a hookworm infection.

## Author Summary

Nematode haemoglobins are fascinating molecules with unusually high affinity for oxygen. This is one example of many unique adaptations that nematodes have acquired to survive in their hosts, as nematode haemoglobin is thought to sequester oxygen to maintain an anaerobic environment, and can break down nitric oxide (NO) and hydrogen peroxide produced by host defences. This study describes the characterization of nematode haemoglobins using a novel monoclonal antibody (anti-Hb) generated against *Anisakis* haemoglobin, which was found to be highly expressed in stage 3 larvae and associated with the excretory-secretary ducts. *Anisakis* haemoglobin is an IgE-binding molecule in infected mice, while *Ascaris* haemoglobin was recognized by IgG but not IgE in human sera. Finally, passive immunization of mice with anti-Hb provided protection against *Nippostrongylus brasiliens* (rodent hookworm), with mice showing reduced worm burden and enhanced Th2 responses, showing that haemoglobin may be a good vaccine target in some nematodes. The monoclonal antibody generated in this study will be useful in further studies to examine the biology of nematode haemoglobins.

## Introduction

Soil-transmitted intestinal nematodes infect more than 1 billion people worldwide, with detrimental effects ranging from anaemia and impaired mental and physical development to potentially fatal intestinal blockages that can result in anywhere from 12 000–135 000 deaths per year [1], [2]. Worm infestations can also increases susceptibility to unrelated infections such as malaria [3], HIV [4] cholera [5] and tuberculosis [6] by skewing immune responses. Evidence exists that nematode infections may also impair vaccine efficacy [7], [8]. The most common soil transmitted nematode species are roundworms (*Ascaris lumbricoides*), whipworms (*Trichuris trichurius*) and hookworms (*Necator americanus* and *Ancylostoma duodenate*) [2], [9].

Nematodes have adapted to survive in particular host species over millions of years, and have evolved numerous mechanisms to adapt to their typically hypoxic ecological niche in the host and survive host defences. One group of molecules, the nematode haemoglobins, are intriguing both from an evolutionary standpoint [10], [11], [12], [13] and because of their unusually high affinity for oxygen [12], [14]. *Ascaris* haemoglobin has been best explored, and is an octameric molecule that binds oxygen nearly 25 000 times more tightly than human haemoglobin [14]. Another high-oxygen affinity haemoglobin was identified in *Pseudoterranova decipiens* [15], a marine nematode that parasitizes seals and is closely related to *Anisakis* species, which can accidentally infect humans. The function of *Ascaris* haemoglobin (and other nematode haemoglobins) is unknown, as it is considered to bind oxygen too tightly to be involved in its delivery, and may rather sequester oxygen to maintain an anaerobic environment [12], [14]. Furthermore, nematode haemoglobin can bind and break down nitric oxide (NO) and hydrogen peroxide, suggesting that it may provide protection against host oxidative defences [12], [14], [16]. Its association with sterols also suggests a potential role in egg production, which requires oxygen [12].

In the present study, a monoclonal antibody (4E8g) was found to recognize highly immunogenic excretory-secretary haemoglobins of both *Anisakis* and *Ascaris*, as well as haemoglobin of the more distantly related murine hookworm *Nippostrongylus brasiliensis*, commonly used in mouse models of nematode infection. Passive immunization of mice with anti-Hb reduced worm burdens after *N. brasiliensis* infection, demonstrating that haemoglobin of some nematodes may be a potential target for new treatments or vaccines against nematode infections.

## Materials and Methods

### Ethics statement

This study was performed in strict accordance with the South African code of practice for laboratory animal procedures, and all mouse experiments were performed according to protocols approved by the Animal Research Ethics Committee of the Health Sciences Faculty, University of Cape Town. Research on the human serum samples was approved by the University of Cape Town Research Ethics committee, and written informed consent was obtained from all participants.

### Preparation of nematode extracts

Live *Anisakis pegreffii* (*A. pegreffii*) larvae (L3) were removed from the gut of parasitized fish (*Thyrsites atun*) obtained at a fish market, and washed in alternating volumes of PBS and acetic acid (4%). Adult *Ascaris lumbricoides* worms were collected after surgical removal at Red Cross Children's Hospital, Cape Town. *Syphacia obvelata* adult worms were collected from mice as previously described [17]. Nematodes were killed by freezing then homogenized in PBS, and the extracts were sonicated, centrifuged and filter sterilized using a 0.20 µm filter (Sartorius, Goettingen, Germany). *Nippostrongylus brasiliensis* extract was made from *N. brasiliensis* L3 by snap freezing larvae in liquid nitrogen, homogenizing the extract and centrifuging to remove debris. *Anisakis* ES products were produced by incubating *Anisakis* larvae at 37°C for 5 days in saline solution. The ES products were then concentrated using Millipore protein concentration tubes with a 5 kDa cut-off (Amicon Ultra). The BCA Protein Estimation Kit (Pierce, Rockford) was used to measure protein concentrations. *Heligosmoides polygyrus* (adult worm) and *Contracaecum* sp (stage L3) extracts were kind gifts from Dr. Rick Maizels (University of Edinburgh, U.K.) and Dr. Shokoofeh Shamsi (Charles Sturt University, Australia) respectively.

### Generation of monoclonal antibodies

A BALB/c mouse was immunized subcutaneously with 20 µg of *Anisakis* extract in Freund's complete adjuvant. At weeks 3 and 6 the mouse was boosted with 20 µg *Anisakis* extract in Freund's incomplete adjuvant. At week 9 the mouse was boosted with 20 µg *Anisakis* extract in PBS, and three days later the mouse was killed and the spleen removed. Isolated lymphocytes were fused with AgX63.653 murine myeloma cells using 50%PEG 6000/15% DMSO in DMEM-20. Fused cells were plated in DMEM-20-HAT at 5×10^5^ cells/ml in 96 well plates and incubated at 37°C. After 7–10 days, supernatant from wells containing clones was tested for *Anisakis*-specific IgG antibody by ELISA. Positive clones were sub-cloned and weaned off HAT by culturing in DMEM-20-HT, followed by culturing in DMEM-20. The positive subclone 4E8g (IgG1 isotype) was chosen for further studies based on its stability and cross-reactivity to *Ascaris lumbricoides.*

### Identification of 4E8g antigen

The Seize X Protein G Immunoprecipitation kit (Pierce, Rockford) was used according to the manufacturer's protocol to isolate the antigens binding to 4E8g in *Anisakis* and *Ascaris* extracts. Eluted proteins were analyzed by SDS-PAGE and immunoblotting using 4E8g. Electrophoresed proteins were stained with GelCode Blue (Pierce, USA) and excised from the gel. Gel slices were destained with 200 mM NH4HCO3∶acetonitrile (Romill, Sigma) 50∶50 until clear. Samples were dehydrated and dessicated with a SpeedVac SC110 (Savant) before reduction with 5 mM Tris-(2-carboxyethylphosphine) (TCEP, Fluka) in 100 mM NH_4_HCO_3_ for 30 minutes at 56°C. Excess TCEP were removed and the gel pieces again dehydrated. Cysteines were carbamidomethylated with 100 mM iodoacetamide (Sigma) in 100 mM NH_4_HCO_3_ for 30 minutes at room temperature in the dark. After carbamidomethylation the gel pieces were dehydrated and washed with 50 mM NH_4_HCO_3_ followed by another dehydration step. Proteins were digested by rehydrating the gel pieces in proteomics grade trypsin solution (20 ng/uL)(Roche) and incubating at 37°C overnight. Peptides were extracted from the gel pieces with 50 µL 0.1% trifluoroacetic acid (TFA) (Sigma). The samples were desiccated and 50 µL water was added, then concentrated to less than 20 µL to remove residual NH_4_HCO_3_

The eluted peptides were spotted using the dried droplet technique with two times 0.5 uL overlay. The matrix was 10 mg/mL α-cyano-4 h-ydroxycinnamic acid (Fluka) with 20 mM NH_4_H_2_PO_4_ (Fluka) in 80% acetonitrile, 0.2% TFA for a final concentration of 5 mg/mL matrix in 40% acetonitrile, 0.1% TFA, 10 mM NH_4_H_2_PO_4_ (Fluka). Mass spectrometry was performed with a 4800 MALDI ToF/ToF (Applied Biosystems). All MS spectra were recorded in positive reflector mode. Spectra were generated with 400 laser shots/spectrum at laser intensity of 3800 (arbitrary units) with a grid voltage of 16 kV. All peptide containing spots were internally calibrated using trypsin autolytic fragments. Database interrogation was performed with the Mascot algorithm using the MSDB database on a GPS workstation (www.matrixscience.com). Search parameters were as follows: Species – all entries, Enzyme – trypsin, Maximim number of missed cleavages - −1, Fixed modifications – carbamidomethyl (C), Variable modifications – oxidation (M), Precursor tolerance – 50 ppm. Protein scores >65 were considered significant. Sequences obtained from MS/MS analysis were analyzed using the BLAST search engine.

### Immunoblotting

For immunoblot analysis, nematode extracts or purified haemoglobins were separated on 10% acrylamide gels and transferred to a HybondC+ nitocellulose membrane (Amersham, Biosciences, UK) at a constant voltage of 300 mA for 1 h. Haemoglobin was detected using 4E8g diluted 1∶2000 in 1% milk/TBS and incubated overnight at 4°C. An alkaline phosphatase-labelled goat anti-mouse IgG1 secondary antibody (Southern Biotech, USA) diluted 1∶1000 in 1% milk/TBS was used with 5-bromo-4-chloro-3-indolyl phosphate-4-nitroblue tetrazolium (Sigma-Aldrich, Germany). For the detection of haemoglobin-specific mouse IgG and IgE, serum collected from *Anisakis*-infected mice in a previous study [18] was used with AP-labelled goat anti-mouse IgG1 (Southern Biotech, USA) and goat anti-mouse IgE (Southern Biotech, USA) as secondary antibodies. For the detection of haemoglobin-specific human IgG, sera were used from individuals with elevated specific IgE to *Anisakis* and *Ascaris* [19]. Anti-*Ascaris* specific IgE ranged from 0.9 kU/L–17.9 kU/L in these subjects while anti-*Anisakis* specific IgE ranged from 0.6 kU/L–16.9 kU/L. Negative control sera had 0 kU/L specific IgE. Antibodies were detected with AP-labelled anti-human IgG and IgE (Sigma).

### ELISA

Binding of 4E8g to mouse blood was measured by measured by ELISA. Whole mouse blood was collected in EDTA to prevent coagulation and used in the ELISA. Sample preparation was based on commercially available mouse haemoglobin ELISA protocols (Abcam). Plates were coated with *Anisakis* extract (positive control) or mouse blood using concentrations from 0.4 ng/ml to 4 mg/ml, and blocked with 4% milk powder/PBS. Monoclonal antibody 4E8g was added overnight at 4°C at a concentration of 30 µg/ml, and detection was carried out using alkaline phosphatase labelled anti-mouse IgG1 (Southern Biotechnology) with P-nitrophenylphosphate substrate (Sigma-Aldrich). Absorbance was measured at 405 nm with 492 nm as a reference wavelength.

### Sequencing of *Anisakis* haemoglobin

Total RNA was extracted from *Anisakis* larvae using the Nucleospin RNA II kit (Macherey-Nagel, Germany) and cDNA was generated using the Transcriptor First Strand cDNA Synthesis Kit (Roche, Germany). Degenerate PCR was performed using primers designed with Primer3 (http://frodo.wi.mit.edu) [20], using highly conserved regions of aligned *Ascaris lumbricoides* and *Pseudoterranova decipiens* haemoglobin sequences. The following forward primers: 5′-CACGTTITITGCGCCAC(A/C)TACGA-3′ and reverse primer: 5′-TGTCCTTGTTT(A/C)ACGAAGAA-3′ were used. PCR products were analysed by agarose gel electrophoresis (1.6%) and fragments of the correct size were excised and purified using the Nucleospin extract II kit (Macherey-Nagel, Germany). Cloning was carried out using the pGEM-T easy cloning system (Promega, USA) with the pGEM-T easy vector and JMIO9 High Efficiency Competent Cells (*E.coli*) for transformation. Plasmid DNA was extracted from the cells using the SV Miniprep DNA Purification Kit (Promega, USA). The presence of the insert of correct size in the plasmid was confirmed by PCR using the pGEM-T easy primers (Sp6 and T7). Plasmids were sequenced by automated sequencing based on the dye-terminator sequencing method at the Molecular and Cellular Biology Department at UCT. Obtained sequences were used to design gene specific primers for 5′ (5′-GAAGACCACGATGGACGAGCCAACC-3′) and 3′ (5′-CACGTCCTCAGCCGTGTAGTTCTCG-3′) rapid amplification of cDNA ends (RACE). Amplification of the 5′ and 3′ regions of the haemoglobin sequence was conducted using the SMART RACE CDNA Amplification Kit (Clontech) with the Primescript Reverse Transcriptase (Takara, Japan). The resulting PCR products were cloned and sequenced as described above. An additional round of RACE-PCR was then performed on the extended sequence using SMARTER-RACE PCR kit with the following primers: 3′ RACE: CCGACGTTCGATTTCTTCGTTGAC, 5′ RACE: GCGAGCAGGATGTTTTGACCTTGT, CTCATGCAATGGTCACGAAC and CGAAGAACTCGTCCTTCTGC. Final gene sequences were analyzed on the ClustalW program (www.ebi.ac.uk/clustalw/) to assess their homology to the known *Ascaris* and *Pseudoterranova* haemoglobin sequences. A phylogenetic tree was constructed using MEGA5 with neighbour joining and the minimum evolution method [21]. Amino acid sequences of the haemoglobins from these nematodes were aligned using MUSCLE (http://www.ebi.ac.uk/Tools/muscle/index.html) and analyzed on Jalview [22].

### Immunohistochemical staining

For histology, specimens of *Anisakis* (L3), *A. lumbricoides* (adult worm), *N. brasiliensis* (adult worms) and small intestines from mice with *N. brasiliensis* infection (day 7 post infection) were preserved in 4% phosphate-buffered formalin overnight, cut in paraffin sections 5 to7 µm, washed in alcohol and pre-blocked with 3% H_2_0_2_. Antigen retrieval was performed for 2 min in 0.1 M Citrate Buffer pH6 in a pressure cooker, sections were blocked with normal serum and Avidin/Biotin using a Vector Blocking Kit (Dako), and then stained with haemoglobin-specific 4/E8g antibody or a mouse IgG1 isotype control (anti-TNP mouse IgG1, a kind gift from Dr Reece Marillier and Dr Jacques van Snick, Ludwig Institute for Cancer Research, Brussels, Belgium). Detection was then carried out using mouse Envision (Dako) with DAB substrate (Dako) and slides were counterstained with Mayers Haematoxylin.

### Treatment of mice with 4E8g

Wildtype BALB/c mice were housed in independently ventilated cages under specific pathogen free conditions in the University of Cape Town Animal Facility. Mice were injected with 200 µg of 4E8g in 200 µl PBS intraperitoneally one day prior to infection with 500 *N. brasiliensis* L3. Control mice were injected with anti-TNP mouse IgG1 isotype control antibody (a kind gift from Dr Reece Marillier and Dr Jacques van Schnick, Ludwig Institute for Cancer Research, Brussels, Belgium). Mice were killed at 12, 24, 48 and 72 hours and 5, 7 and 10 days post-infection, and intestines and lungs were collected for worm counts. Whole lungs were collected at 12, 24, 48, 72 and 120 hours post-infection, cut into 2–5 mm pieces and placed in a sterile gauze. The gauze was placed in a 15 ml Falcon tube filled with 0.9% NaCl and left overnight at 37°C allowing the worms to migrate out of the lungs. Intestines were collected at days 3, 5, 7 and 10 in 0.9% NaCl and incubated for 3 hours at 37°C for worm counts. The following day the gauze with lung was removed and worms were counted under a dissecting microscope. Mesenteric lymph nodes were collected and single cell suspensions were restimulated with anti-CD3 for 72 hours. Cell supernatants were then collected for cytokine ELISAs, as previously described [18]. Mouse mast cell protease (MMCP-1) was quantified in serum from infected mice as previously described [18].

### Statistics

Data is given as mean ± SEM. Statistical analysis was performed in GraphPad Prism (Prism software; http://www.prism-software.com) using the unpaired Student's *t* test(*, p≤0.05; **, *p*≤0.01; ***, p≤0.001).

## Results

### Monoclonal antibody 4E8g binds nematode haemoglobin

**Figure 1:**
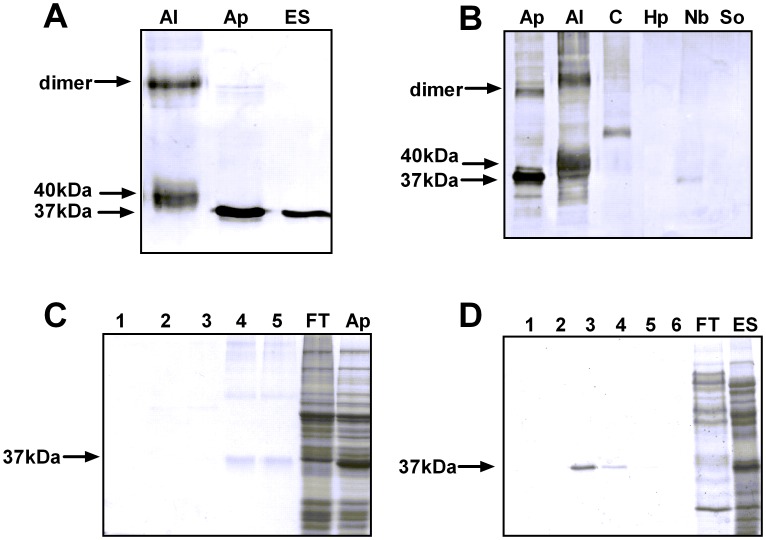
Antigen recognition by monoclonal antibody 4E8g. **A**) 4E8g recognizes a 40 kDa protein in *A. lumbricoides* extract (Al) and a 37 kDa protein in *A. pegreffii* somatic extract (Ap) and excretory-secretory proteins (ES) **B**) Antigen recognition in different nematode extracts. Ap, *Anisakis pegreffii*; Al, *Ascaris lumbricoides*; C, *Contracaecum* sp.; Hp, *Heligosomoides polygyrus*; Nb, *Nippostrongylus brasiliensis*; So, *Syphacia obvelata*. 4E8g antigen could be successfully purified from both (**C**) somatic and (**D**) excretory-secretory extracts of *Anisakis* by immunoaffinity spin columns and was identified as *Anisakis* haemoglobin. 1–6 = elution fractions 1–6, FT, flowthrough of spin column, Ap, *Anisakis pegreffii*, ES, excretory-secretory proteins.

Using immunoblotting, the monoclonal antibody 4E8g was found to bind to a 37 kDa protein present in both somatic and excretory-secretory extracts of *A. pegreffii* L3, and a 40 kDa protein (as well as an 80 kDa protein presumed to be a dimer [23]) present in *A. lumbricoides* worm whole protein extract (Figure 1A). 4E8g also recognized a 37 kDa protein in *N. brasiliensis* L3 and a higher MW protein in *Contracaecum* spp. L3, but did not recognize proteins in *H. polygyrus* or *S. obvelata* worm extracts, indicating that the antibody is cross-reactive among several nematodes but not all (Figure 1B). An immunoprecipitation kit was then used to identify the antigens bound by 4E8g. Affinity-based purification of the antigen was very effective, with a distinct 37 kDa antigen visible following elution of 4E8g binding proteins in both somatic *Anisakis* extract and *Anisakis* ES (Figure 1C and D). Immunoprecipitation to purify the 4E8g binding protein from both *A. lumbricoides* extract resulted in elution of a 40 kDa band respectively (data not shown). Trypsin digestion and MS/MS analysis was used to identify the eluted proteins. The *Ascaris* 40 kD protein matched with high confidence with a score of 420 (protein score >65 is significant; p<0.05) to pig roundworm (*Ascaris suum*) haemoglobin. *Ascaris suum* and *Ascaris lumbricoides* are extremely closely related both morphologically and biochemically [24]. There was no exact match for the eluted *Anisakis* protein, which was not in the database, but PMF showed a match with the haemoglobin protein of *Pseudoterranova decipiens* with low significance (score of 61). In addition, MS/MS followed by a MSBLAST search showed a high peptide match (HMFEHYPHMR) to *Pseudoterranova decipiens* haemoglobin. *Pseudoterranova* and *Anisakis* are closely related marine nematodes belonging to the same family, indicating that 4E8g recognizes *Anisakis* haemoglobin.

### Recognition of *Anisakis* and *Ascaris* haemoglobin by mouse and human serum

**Figure 2:**
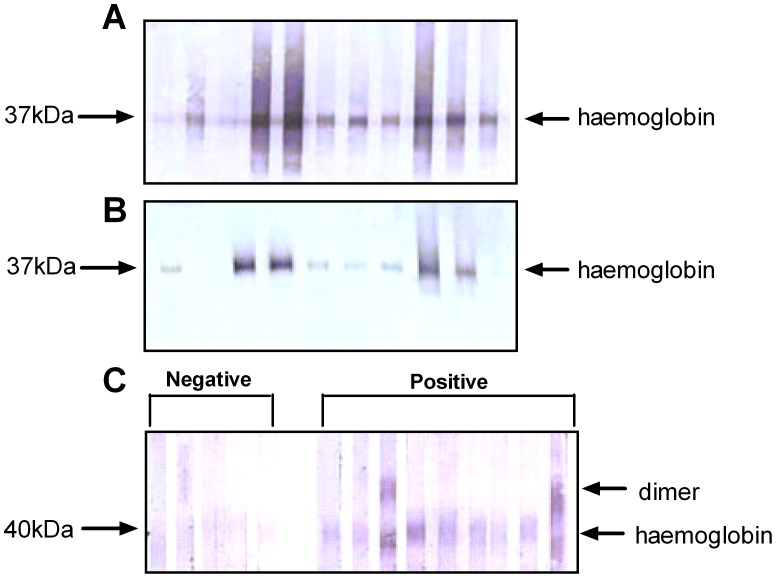
Immunoblotting with purified haemoglobin. **A**) Specific IgG1 against purified *Anisakis* haemoglobin in serum from mice infected at week 0 with two live *A. pegreffii* L3 and re-infected at week 8 with 2 L3. Serum was collected at week 11. Each lane represents one individual mouse. **B**) Specific IgE against purified *Anisakis* haemoglobin in mouse serum, collected as in (A). Each lane represents one individual mouse. **C**) Specific IgG against purified *Ascaris* haemoglobin in human serum samples that were negative (0 kU/L specific IgE, CAP-RAST) or positive (0.9–17.9 kU/L specific IgE, CAP-RAST) for *Ascaris* specific antibodies. Each lane represents one individual.

*Anisakis* and *Ascaris* haemoglobins were purified using the 4E8g antibody (anti-Hb) with the Seize X Protein G immunoprecipitation kit, electrophoresed and transferred onto nitrocellulose membranes. Immunoblotting with serum from mice that had been infected with live *Anisakis* larvae showed that mice had generated both specific IgG (Figure 2A) and specific IgE (Figure 2B) to *Anisakis* haemoglobin. IgG from most of the mice also recognized *Ascaris* haemoglobin by cross-reactivity. Interestingly, none of the mouse IgE recognized *Ascaris* haemoglobin (data not shown). In addition, immunoblotting was carried out using sera from subjects who had tested positive for specific IgE against *Ascaris* and *Anisakis*. Specific IgG antibodies against *Ascaris* haemoglobin seemed to be present in the majority of *Ascaris*-positive subjects (Figure 2C), while specific IgE against haemoglobin could also not be detected in these human sera (data not shown). *Anisakis* haemoglobin was not recognized by any of the sera.

### Sequencing of *Anisakis* haemoglobin

**Figure 3:**
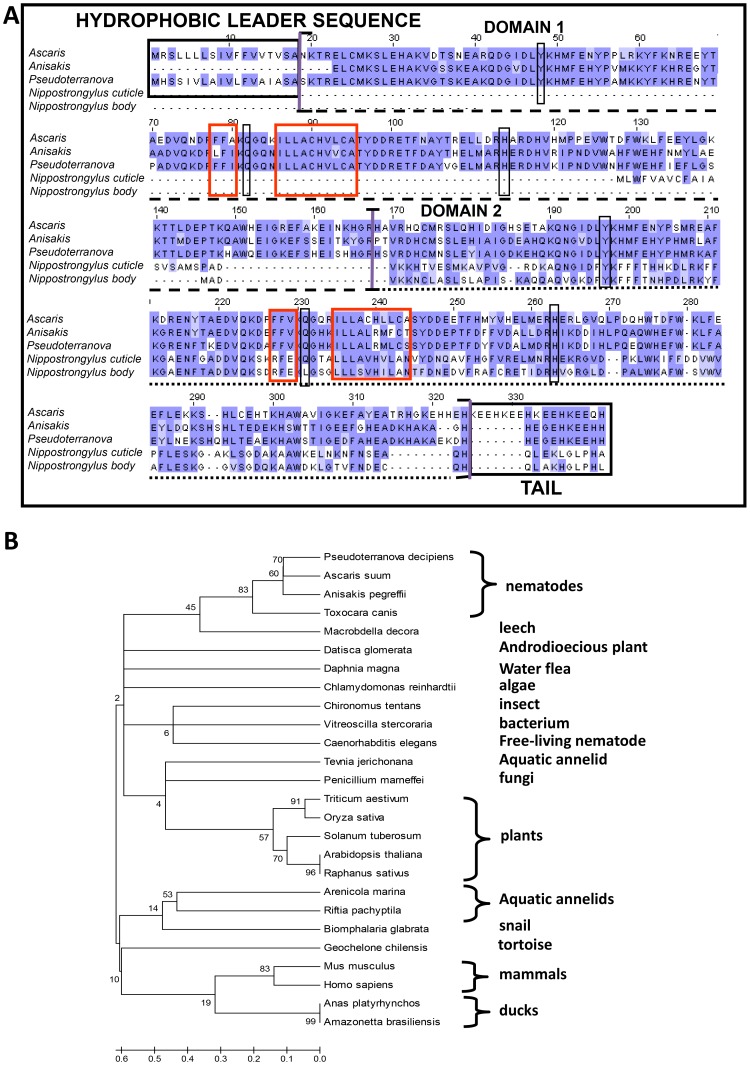
Amino acid alignment of *Anisakis* haemoglobin with haemoglobins of *Ascaris*, *Pseudoterranova* and *Nippostrongylus brasilensis*, and phylogenetic analysis. **A**) The cDNA sequence of *Anisakis* haemoglobin was obtained by degenerate PCR and RACE-PCR (accession number: JX860676) and the amino acid sequence was deduced. Alignment of sequences was carried out using MUSCLE (http://www.ebi.ac.uk/Tools/muscle/index.html). The hydrophobic leader portion (sequence not obtained in *Anisakis*) and the C-terminal tail (important for assembly of octamers in *Ascaris*) are boxed in black, while hydrophobic haem-binding regions are boxed in red. The haemoglobin protein consists of two homologous domains, illustrated by dashed and dotted lines. The B10 tyrosine, E7 distal glutamine and F8 proximal histidine, important in binding of oxygen, are conserved between the three nematodes and are marked with boxes. **B**) A range of haemoglobin sequences across different species was obtained by BLAST search. The evolutionary history was inferred using the Minimum Evolution method [21]. The bootstrap consensus tree inferred from 2000 replicates is taken to represent the evolutionary history of the taxa analyzed. Branches corresponding to partitions reproduced in less than 50% bootstrap replicates are collapsed. The tree is drawn to scale, with branch lengths in the same units as those of the evolutionary distances used to infer the phylogenetic tree. The evolutionary distances were computed using the Maximum Composite Likelihood method and are in the units of the number of base substitutions per site. The ME tree was searched using the Close-Neighbor-Interchange (CNI) algorithm at a search level of 0. The Neighbor-joining algorithm was used to generate the initial tree. The analysis involved 26 nucleotide sequences. Codon positions included were 1st+2nd+3rd+Noncoding. All positions containing gaps and missing data were eliminated. There were a total of 64 positions in the final dataset. Evolutionary analyses were conducted in MEGA5.

Degenerate PCR resulted in a partial nucleotide sequence of 428 bp, which was used to design gene specific primers for 5′ and 3′ rapid amplification of cDNA ends. Despite repeated RACE reactions, the sequence for the initial 22 amino acids in the 5′ region was not obtained. However, complete sequencing at the 3′ end demonstrated that *Anisakis* haemoglobin is similar to *Pseudoterranova* haemoglobin and lacks the four –HKEE repeats of *Ascaris* haemoglobin that promote the assembly of tetramers [12] (Figure 3A). Like both *Ascaris* and *Pseudoterranova* haemoglobins, the *Anisakis* haemoglobin consists of two similar domains, each with a haem binding site, and has the B10 tyrosine, E7 glutamine and F8 histidine that are important for high oxygen avidity [12]. 4E8g also recognized the more distantly related *N. brasiliensis* haemoglobin (Figure 1B). Unlike ascarid haemoglobins, which contain two similar domains, *N.brasiliensis* haemoglobin contains one domain which occurs in two isoforms, a body globin isoform and a cuticular globin isoform [23]. This is thought to be due to gene duplication event similar to the one that resulted in the two domain gene of ascarid haemoglobins [23]. This one-domain protein tends to dimerize to a 37 kDa protein, as seen in Figure 1B, and shows high similarity to the second domain of *Anisakis* haemoglobin, explaining why the monoclonal antibody is cross-reactive (Figure 3A). A phylogenetic tree assembled using published haemoglobin sequences from various organisms shows that *Anisakis* haemoglobin branches off with *Pseudoterranova* haemoglobin, but is also closely related to *Ascaris* haemoglobin (Figure 3B).

### Location of haemoglobin in the excretory-secretory apparatus in *Anisakis*, *Ascaris* and *Nippostrongylus brasiliensis*

**Figure 4:**
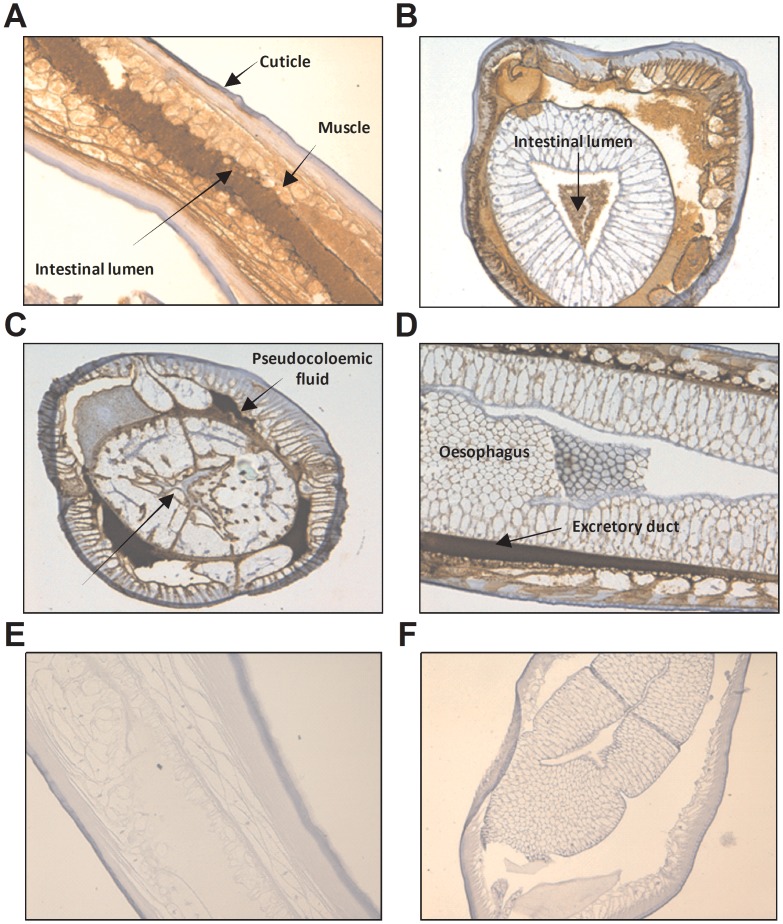
Expression of haemoglobin in *Anisakis pegreffii*. **A–D**) *A. pegreffii* L3 collected from *Thyrsites atun* were formalin fixed and stained with 4E8g (Anti-Hb). **E–F**) *A. pegreffii* L3 sections were stained with isotype control antibody (mouse IgG1). Detection was carried out using the anti-mouse IgG detection system with DAB. Haemoglobin is stained in brown. Counterstaining was performed with haemotoxylin. All photos taken at 200× magnification.

**Figure 5:**
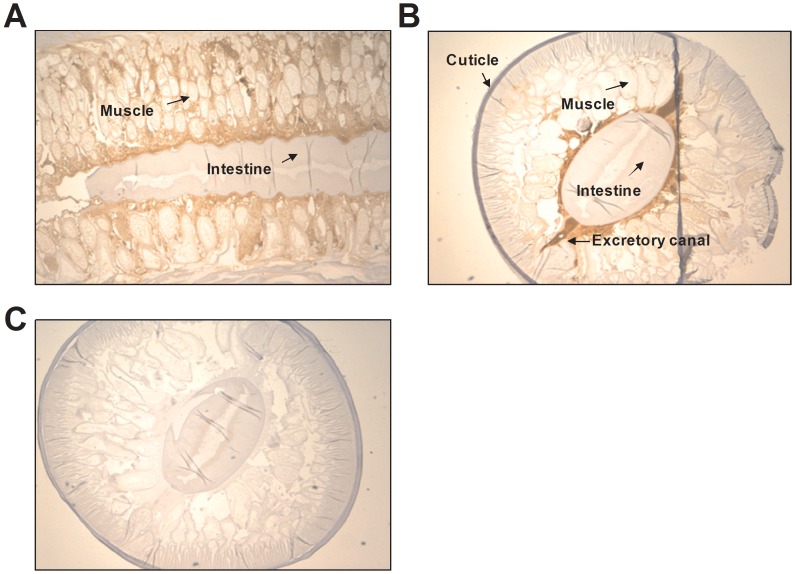
Expression of haemoglobin in *Ascaris lumbricoides*. **A**) Longitudinal sections and **B**) cross sections of a formalin-fixed *A. lumbricodes* adult worm was stained with 4E8g (Anti-Hb) and detected with the anti-mouse IgG detection system with DAB. **C**) *A.lumbricoides* section stained with isotype control antibody (mouse IgG1). Haemoglobin is stained in brown. Counterstaining was performed with haemotoxylin. All photos taken at 40× magnification.

**Figure 6:**
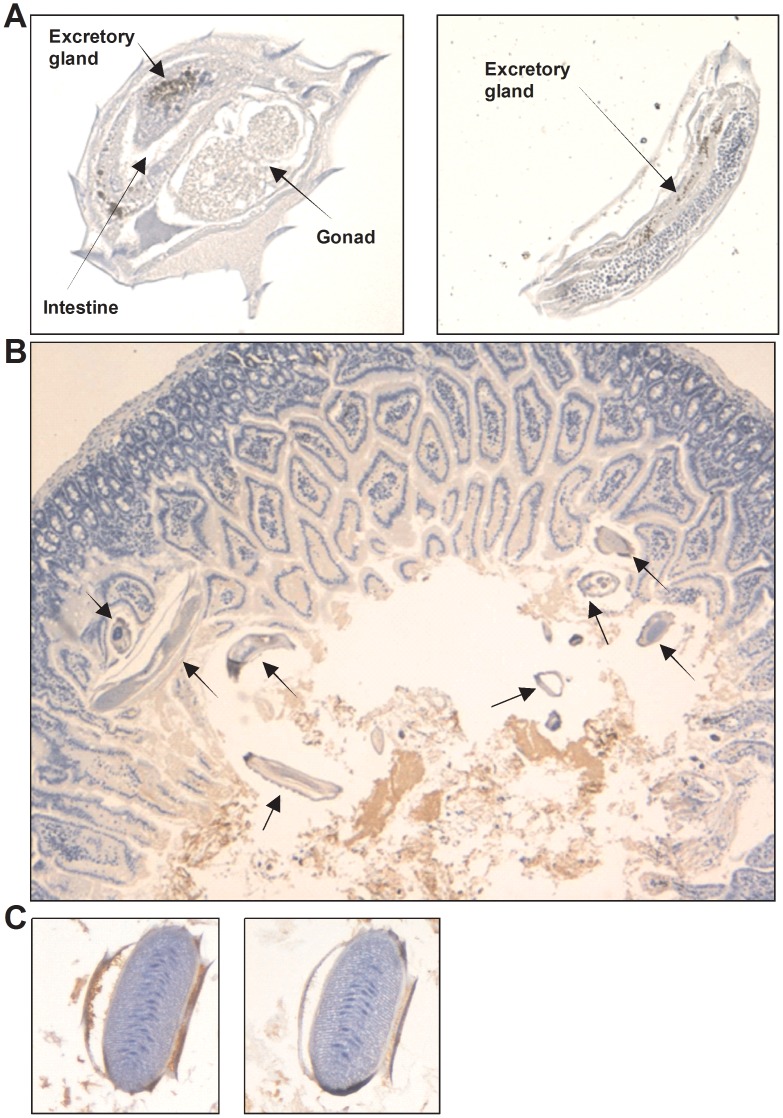
Expression of haemoglobin in *Nippostrongylus brasiliensis*. **A**) *N. brasilensis* adult worms isolated from mouse guts were formalin fixed and stained with 4E8g (Anti-Hb). Detection was carried out using the anti-mouse IgG detection system with DAB. Haemoglobin is stained in brown. Counterstaining was performed with haemotoxylin. 400× magnification. **B**) Small intestines of mice infected with *N. brasiliensis* (Day 7 post-infection) were formalin fixed, stained with Anti-Hb and counterstained with haemotoxylin. *N. brasiliensis* worms are indicated by arrows. 40× magnification. **C**) *N.brasilensis* larvae in mouse intestines stained with 4E8g (left) and isotype control antibody (mouse IgG1)(right).

The monoclonal antibody 4E8g allowed for immunohistochemical staining to identify the location of haemoglobin expression in *Anisakis*, *Ascaris* and *N. brasiliensis* (Figures 4–6). Staining of *Anisakis* sections confirmed immunoblotting results, indicating that *Anisakis* haemoglobin is an excretory-secretary protein (Figure 4). Haemoglobin is highly concentrated in the intestinal lumen of the *Anisakis* larvae (Figure 4A,B), but is also found in the surrounding muscle cells and just below the cuticle. The staining demonstrates that haemoglobin is an ES product that is expressed in the gut and passes via the excretory glands into the environment (Figure 4C,D). Sections stained with isotype control antibody did not show any staining in these areas (Figure 4 E–F). *Ascaris* haemoglobin also appears to be expressed from the gut through excretory cells (Figure 5) and is also found within muscle cells. Similarly, in *N. brasiliensis* worms, haemoglobin was associated with excretory glands near the intestine (Figure 6A, B), suggesting that this nematode species may also secrete haemoglobin. Staining of haemoglobin in cross-sections of *N. brasiliensis* worms that were present in infected mouse small intestine also demonstrated the presence of haemoglobin in peripheral areas such as the fluid-filled median zone of the cuticle (Figure 6B, C).

### Passive immunization of mice with anti-haemoglobin antibody reduces worm burdens in *N. brasiliensis* infection

**Figure 7:**
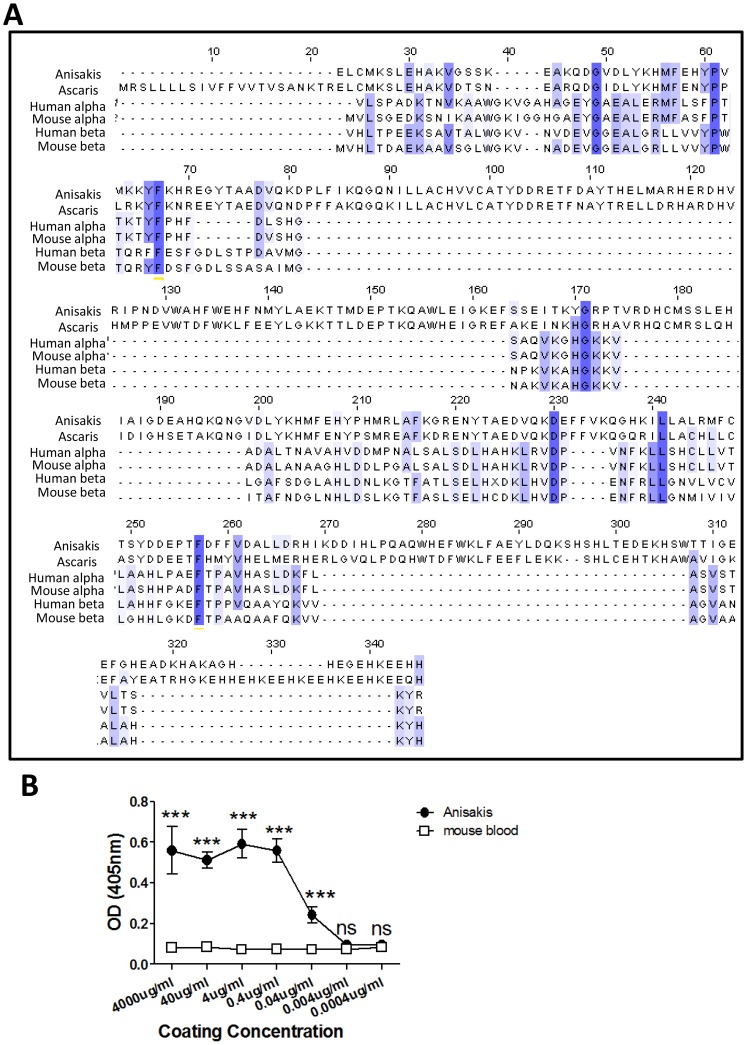
Monoclonal antibody 4E8g does not recognize mouse haemoglobin. **A**) Alignment of sequences of *A.pegreffii*, *A.lumbricoides*, human and mouse haemoglobin was carried out using MUSCLE (http://www.ebi.ac.uk/Tools/muscle/index.html). Percentage agreement to consensus sequence is coloured as follows: mid-blue: >80%, light blue: >60%, light grey >40%, white </ = 40%. **B**) Binding of 4E8g to mouse blood was measured by ELISA. Whole mouse blood was collected in EDTA to prevent coagulation, based on commercially available mouse haemoglobin ELISA protocols. Plates were coated with *Anisakis* extract (positive control) or mouse blood using concentrations from 0.4 ng/ml to 4 mg/ml and incubated with monoclonal antibody 4E8g to assess binding. Detection was carried out using alkaline phosphatase labelled anti-mouse IgG1 with P-nitrophenylphosphate substrate.

**Figure 8:**
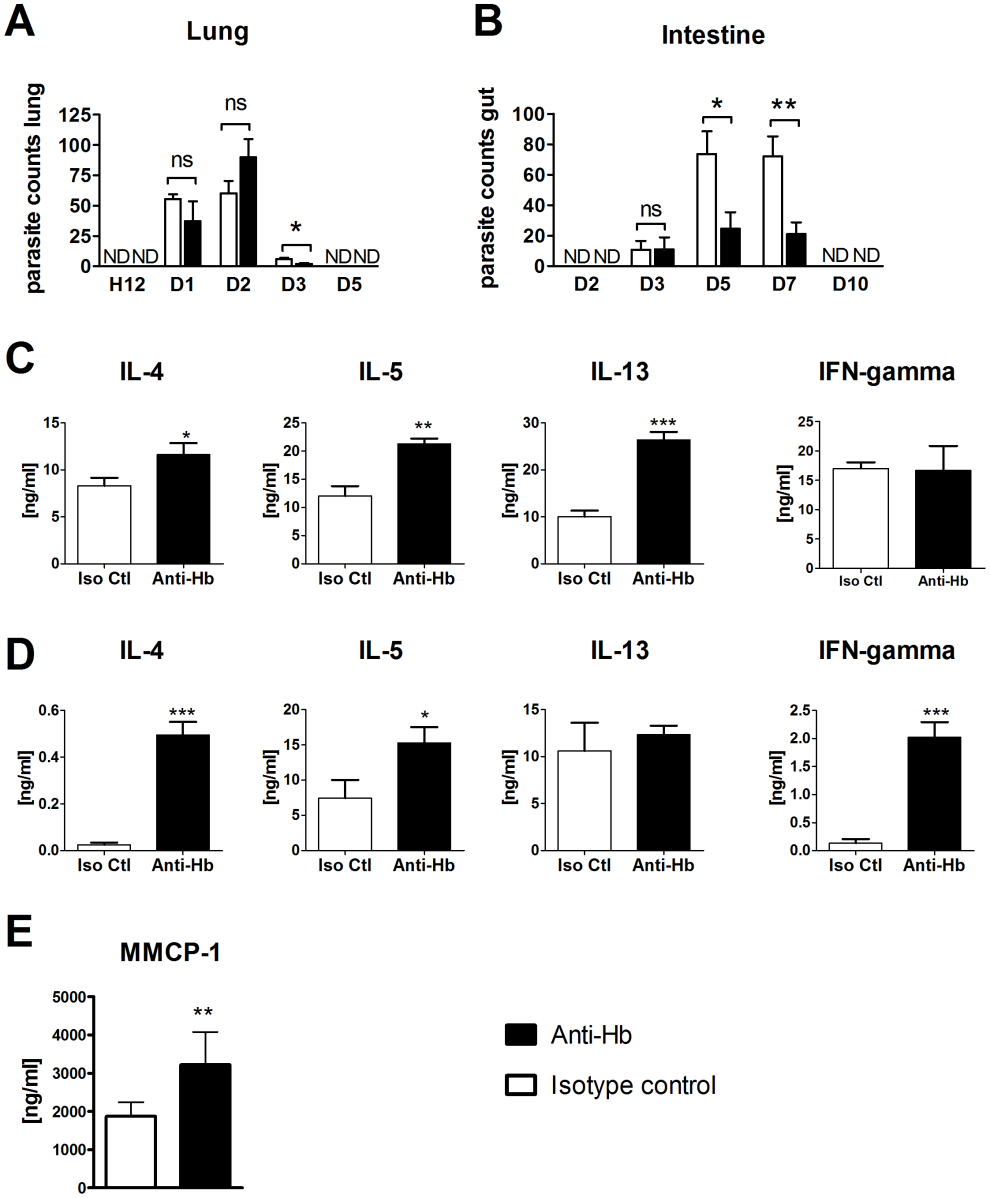
Passive immunization with Anti-Hb reduces worm burdens in *Nippostrongylus brasiliensis* infected mice. Mice (n = 5–8) were injected with 200 µg of 4E8g (Anti-Hb) at day −1 and infected with 500 *N. brasiliensis* L3 at Day 0. **A**) Worm burdens in the lung at different timepoints post infection. Bars represent the mean and error bars show standard error of the mean (SEM). **B**) Worm burdens in the intestine at different timepoints post infection. Bars represent the mean and error bars show SEM. **C–D**) Mesenteric lymph node cells from individual mice at day 7 (n = 5–8) were restimulated with **C**) anti-CD3 or **D**) *N. brasiliensis* somatic extract for 72 hours and cytokines were measured in the supernatant by ELISA. Bars represent the mean and error bars show SEM.**E**) Mouse mast cell proteases in serum at day 7. Statistical analysis was performed in GraphPad Prism using the unpaired Student's *t* test (*, p≤0.05; **, *p*≤0.01; ***, p≤0.001).

Nematode and vertebrate haemoglobins are very dissimilar [23] (Figure 7A), with nematode haemoglobins showing only 10–15% homology to vertebrate haemoglobins [13] 4E8g was shown not to bind mouse haemoglobin by ELISA and western blotting (Figure 7B). To determine whether nematode haemoglobin could be targeted for immunization strategies, mice were injected with 4E8g one day prior to *N. brasiliensis* infection. Parasite burdens were counted in the lungs and intestine at different timepoints post infection. *N. brasiliensis* L3 migrate to the lungs after injection and moult into L4 larvae between 19–32 hours [25] The L4 remain in the lung till approximately 50 hours post infection before penetrating the alveoli, being coughed up and swallowed into the intestine. In the intestine they moult into adult worms at about 90–108 hours post infection. The number of parasites in the lungs was similar in anti-Hb and isotype control treated groups at 24 h and 48 h post infection (Figure 8A). Parasite numbers were low in the lungs in both groups at 72 h due to migration out of the lungs, although in anti-Hb treated mice the parasite numbers were significantly lower. In the intestine, equivalent worm burdens were found in both groups of mice at 72 h post infection. However at both day 5 and day 7 post infection, worm burdens were significantly reduced in anti-Hb treated mice. Protection was associated with increased levels of MMCP-1, a marker of mast cell degranulation, in the serum (Figure 8B) and increased production of Th2 cytokines by restimulated mesenteric lymph node cells (Figure 8 C–F). Together these data suggest that anti-Hb primarily confers protection against the adult worm, either directly or by priming the immune system to increase expulsion or destruction of the parasites.

## Discussion

Nematode haemoglobins have unusually high oxygen affinity and a range of studies indicate that they may represent an important component of parasite adaptation to co-existence with the host. Haemoglobin sequestering of oxygen has been suggested to aid the parasite in maintaining a locally anaerobic environment while their ability to break down nitric oxide (NO) and hydrogen perdoxide produced by innate immune cells would also aid parasite survival [12], [14]. *Ascaris* haemoglobin may also play a role in egg production, as oxygen is required for sterol synthesis [12]. As such, parasite haemoglobins represent an attractive target for vaccines. In the present study, a monoclonal antibody was generated against a nematode haemoglobin, and conferred protection against *N. brasiliensis* in a mouse model.

While cheap and effective drugs exist for the treatment of nematode infections, rapid re-infection is a frequent problem, and drug resistance is increasing among nematodes of livestock [2]. Understanding host protective responses against nematodes is critical for the design of effective vaccines for humans or livestock [26]. Expulsion of nematode parasites is typically considered to be dependent on T cell mediated immunity and production of Th2 cytokines, particularly IL-13 [27]. However, increasing evidence shows that antibodies also play a protective role [27], [28], [29], [30]. Many studies using a wide range of helminths have shown that transfer of immune sera or purified parasite specific IgG can be protective, depending on the species and the quality of antibody used [28], [30]. Highly specific, affinity-matured antibodies that arise after multiple infections are required for optimal protection. When B cell deficient mice were able to effectively expel *N. brasiliensis* in both primary and secondary infections, it was concluded that antibodies were unlikely to play a significant role in protection against this species, although the authors did note that immune serum could contribute to protection [31]. In the present study, passive immunization of *N. brasiliensis* infected mice with anti-Hb antibody significantly reduced worm burdens, indicating that while antibodies may be redundant in clearing infection, they can certainly play a protective role, especially when highly specific. After initial experiments demonstrated protection, kinetic experiments were performed in order to determine at which stage of infection the anti-Hb had an effect, since *N.brasiliensis* matures as it migrates through the host and the different stages may require different mechanisms of immunity [32]. Similar numbers of larvae were found in the lungs of treated and untreated groups in the early stages post infection, whereas worm counts in the intestine were significantly lower in the anti-Hb treated mice. This suggests that anti-Hb responses provide protection against the adult intestinal-dwelling stage of the helminth life cycle. Although previous authors did not find expression of globin cDNA in *N. brasiliensis* pre-infection L3 larvae using northern blotting [23], we did detect the globin in extract derived from L3 using 4E8g, which may be a more sensitive method of detection. However since globin cDNA was readily detected by the same authors in parasite stages isolated from the mammalian host, this suggests that expression of *N. brasiliensis* globins is higher in adults, and this may explain why passive immunization with 4E8g was effective only against the adult stage. It was suggested that the increased in size after moulting, the tendency of the larger adult nematodes to lie further into the lumen, where the partial pressure of oxygen is lower, and the advent of egg production all lead to an increased requirement for oxygen that may increase the need for globins [23]. Furthermore, the two *N. brasiliensis* isoforms were found to be differentially expressed, with the body isoform expressed in all stages within the host but the cuticular isoform only expressed after the L4-adult moult [23]. It is therefore possible that the protective effect of 4E8g specifically involves the cuticular isoform of *N. brasiliensis* globin.

It is possible that the anti-haemoglobin antibody acts by recruiting immune cells such as basophils, eosinophils, mast cells and macrophages to attack the parasite. Fcγ cross-linking results in the release of cytokines, chemokines, proteases and free radicals that can enhance immunity and damage the parasite [28]. An increase in Th2 cytokine production and mast cell proteases was observed, indicating that the protective effect could be due to immune system activation. Previously it was shown that passive sensitization with IgG1 antibodies increased allergic inflammation, which has similar characteristics to helminth induced inflammation, possibly by priming mast cells to degranulate after subsequent exposure to antigen [33], [34]. Activated mast cells, basophils and eosinophils release a variety of cytokines and chemokines that can stimulate immune responses, including IL-4 and IL-13, which promote Th2 differentiation and mediate Th2 effector responses, respectively (Metcalfe 2008). Since mast cell proteases, an indicator of mast cell degranulation, were increased in passively immunized mice, this could explain why Th2 responses increased and worm burdens decreased after treatment. However, as hookworms feed on the blood of the host, it is also possible that they ingested anti-Hb antibody, which could then directly bind to haemoglobin in the intestinal lumen, perhaps interfering with its function. Like ascarid haemoglobins, *N. brasiliensis* globins have oxygen affinities 100-fold higher than haemoglobins of their hosts [35], which is thought to help them survive in their anaerobic environment [23]. As nematode haemoglobin from *Ascaris* has been shown to bind NO, it would be interesting to determine whether *N. brasiliensis* haemoglobin has the same property, and if blocking the haemoglobin would thus affect parasite survival by inhibiting its ability to detoxify NO.

It was also shown for the liver fluke, *Fasciola hepatica*, that vaccination with haemoglobin induced protection against infection in cattle [36]. Efficacy was further enhanced by combining the liver fluke haemoglobin with another antigen, cathepsin L2. This combination vaccine was as effective as some flukicides, and not only reduced pathology and fluke growth, but also had a direct effect on egg production, which requires oxygen in this species. Variable protection was also obtained when vaccinating cattle against *Ostertagia ostertagi* with globin enriched from the parasite, with two out of four trials showing some protection [37]. Depending on the expression and function of haemoglobin within a particular parasite species, haemoglobin may therefore be a useful vaccine candidate, particularly in combination with other proteins.

Recently, *Ascaris* haemoglobin was purified by chromatography from pseudocoleomic fluid of *Ascaris suum* and used to vaccinate pigs, after which they were challenged with *Ascaris suum* eggs [38]. Vaccinated pigs showed no difference in faecal egg outputs or worm burdens, but had an increase in white spots on the liver, previously associated with growing resistance to parasite infection [38], [39]. This study also found that haemoglobin was not detectable at the protein level and barely detectable at the transcriptional level in freshly hatched L3 larvae, which may explain why the haemoglobin vaccination was not protective in *Ascaris* infection. In contrast to *Ascaris*, *Anisakis* L3 strongly express haemoglobin, which is therefore an interesting difference between the two species.

Sequencing of *Anisakis* haemoglobin showed that it is most similar to haemoglobin of the closely related marine nematode, *P. decipien*, although haemoglobins of both these nematodes are similar to that of *Ascaris*. Both *Ascaris* and *Pseudoterranova* haemoglobins were found to be extremely oxygen avid and consist of two globin domains followed by a short COOH- terminal tail. The tail of *Ascaris* has four direct repeats of His-Lys-Glu-Glu (HKEE), whereas the *Pseudoterranova* tail has only one HKEE repeat and is 7 amino acids shorter than that of *Ascaris* [40]. In both nematodes the tails are involved in octamer assembly and stability, but the differences in sequence are thought to imply a different mode of oligermisation [12], [41]. Immunoblotting with serum from infected mice demonstrated that IgG antibodies, but not IgE antibodies, were cross-reactive between *Anisakis* and *Ascaris*. IgE antibodies against *Ascaris* haemoglobin were also not detected in human sera, although IgG antibodies were present. This may suggest that *Anisakis* but not *Ascaris* haemoglobin is an IgE-binding protein, which should be investigated in patients with gastroallergic anisakiasis. As neither IgE nor IgG antibodies were detected in the human sera of subjects who had *Anisakis* specific IgE and were occupationally exposed to *Anisakis* proteins, it would also be interesting to see whether live infection with *Anisakis* is required for the development of antibodies to *Anisakis* haemoglobin. This may reveal whether antibodies against *Anisakis* haemoglobin are a useful diagnostic marker of infection. Measurement of anti-Hb antibodies in pigs by indirect ELISA was recently shown to be a reliable means of diagnosing ascariasis in pigs, with higher sensitivity than faecal examination (99.5% versus 59.5% at week 7 and 100% verus 68.4% at week 14) [42]. Detection of IgG antibodies against haemoglobin in human sera could therefore also be investigated as a more sensitive and convenient method of establishing infection with *A. lumbricoides*.

In conclusion, we have generated a specific monoclonal antibody, 4E8g, against *Anisakis* haemoglobin, that can also be used to detect *Ascaris* and *N. brasiliensis* haemoglobin, and may be useful in the detection of haemoglobins from other nematode species that have not yet been tested. 4E8g may be useful in further studies to examine the role and function of nematode haemoglobins and in diagnostic assays for nematode infection. Addtionally 4E8g further demonstrates the potential for anti-haemoglobin antibodies in protecting against certain helminth infections.
